# Supplementary material for: Development of chemokine network inhibitors using combinatorial saturation mutagenesis
Source: Commun Biol. 2025 Apr 3;8:549. doi: 10.1038/s42003-025-07778-6 (PMC11969024; doi:10.1038/s42003-025-07778-6)
Supplement: Supplementary file 4 — Supplementary Data 2 [file 42003_2025_7778_MOESM4_ESM.zip › SupplementaryData3/scripts/xattune_ic50.tar!/./script/analysis/JK/JK_R40_E85_ATHERO_HD2_ATC_plot.pdf]

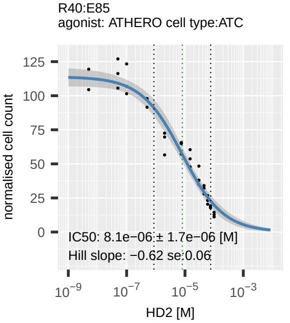
R40:E85
agonist: ATHERO cell type:ATC
normalised cell count
125
100
75
50
25
0
IC50: 8.1e−06 ± 1.7e−06 [M]
Hill slope: −0.62 se:0.06
10^{-9}
10^{-7}
10^{-5}
10^{-3}
HD2 [M]
